# Laparoscopic versus open liver resection for huge hepatocellular carcinoma (≥ than 10 cm): a retrospective analysis from a high-volume referral center

**DOI:** 10.1007/s00464-024-11091-4

**Published:** 2024-08-27

**Authors:** Gianluca Cassese, Ho-Seong Han, Boram Lee, Hae Won Lee, Jai Young Cho

**Affiliations:** 1 Department of Clinical Medicine and Surgery, Division of HBP Minimally Invasive and Robotic Surgery, Transplantation Service, Federico II University Hospital, Naples, Italy; 2 Department of Surgery, Seoul National University College of Medicine, Seoul National University Bundang Hospital, Seoul, South Korea; 3 Department of Health Sciences, University of Eastern Piedmont, Alessandria, Italy

**Keywords:** Huge hepatocellular carcinoma, Hepatocellular carcinoma, Laparoscopic liver resection, Minimally invasive liver surgery

## Abstract

**Background:**

There is still poor evidence about the safety and feasibility of laparoscopic liver resection (LLR) for huge (> 10 cm) hepatocellular carcinomas (HCC). The aim of this study was to assess the short- and long-term outcomes of LLR versus open liver resection (OLR) for patients with huge HCC from real-life data from consecutive patients.

**Methods:**

Data regarding all consecutive patients undergoing liver resection for huge HCC were retrospectively collected from a Korean referral HPB center. Primary outcomes were the postoperative results, while secondary outcomes were the oncologic survivals.

**Results:**

Sixty-three patients were included in the study: 46 undergoing OLR and 17 LLR. Regarding postoperative outcomes, there were no statistically significant differences in estimated blood loss, operation time, transfusions, postoperative bile leak, ascites, severe complications, and R1 resection rates. After a median follow-up of 48.4 (95% CI 8.9–86.8) months, there were no statistically significant differences in 3 years OS (59.3 ± 8.7 months vs. 85.2 ± 9.8 months) and 5 years OS (31.1 ± 9 months vs. 73.1 ± 14.1 months), after OLR and LLR, respectively (*p* = 0.10). Similarly, there was not a statistically significant difference in both 3 years DFS (23.5% ± 8.1 months vs. 51.6 ± months) and 5 years DFS (15.7 ± 7.1 months vs. 38.7 ± 15.3 months), respectively (*p* = 0.13), despite a potential clinically significant difference.

**Conclusion:**

LLR for huge HCC may be safe and effective in selected cases. Further studies with larger sample size and more appropriate design are needed to confirm these results.

**Graphical abstract:**

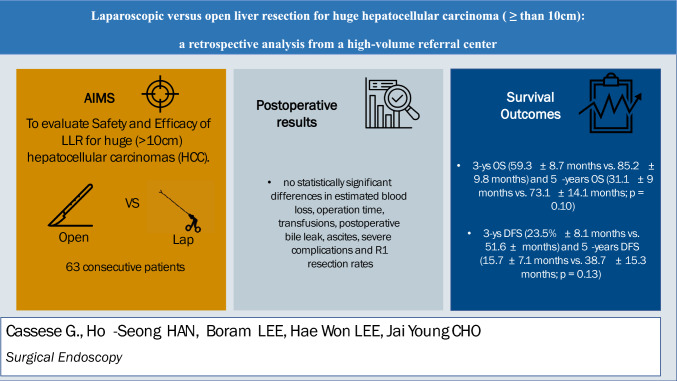

Hepatocellular carcinoma (HCC) represents the most common primary liver malignancy, accounting for the seventh most common cancer worldwide and the third leading cause of cancer-related death [1]. Despite the tremendous medical advances, HCC prognosis is still poor, not exceeding the 5 years overall survival rates (OS) of approximately 20% [2]. Nonetheless, early-staged HCC can benefit from surgical therapy, leading up to 50–70% of 5 years OS [3]. Thus, surgery represents the cornerstone treatment for HCC, including both liver transplantation (LT) and liver resection (LR) [4]. LT aims to treat both HCC and underlying chronic liver disease, but must face the problem of organ shortage, with a consequent risk of dropout from waiting list and tumor progression [5]. Thus, LT is mainly reserved for patients who are not candidates for LR due to impaired liver function or for patients with negative prognostic factors on specimen examination after a previous resection [6]. Accordingly, LR still represents the most performed treatment for early stages. Furthermore, the Milan criteria by Mazzaferro et al. restrict LT in adults to patients with tumor smaller than 5 cm, not more than three and each one not exceeding 3 cm, without angioinvasion, without extrahepatic involvement [7].

Previous data have shown that often HCC patients are often diagnosed at symptomatic and advanced disease stage, with large tumors (> 5 cm) or huge HCC (defined as lesions bigger than 10 cm) [8]. Such huge HCC cannot benefit from LT [9]. Similarly, they are not suitable for thermal ablation in the light of the impossibility to achieve complete tumor necrosis of large lesions [10]. However, according to current guidelines, patients with a solitary HCC and preserved liver function may benefit from liver resection, when preserving a sufficient FRL [11]. Indeed, an extended hepatectomy may be required for such cases, carrying out a non-negligible risk of postoperative morbidity and mortality, mainly related to post-hepatectomy liver failure (PHLF) [12, 13]. Nonetheless, LR has shown to provide significant survival benefits when compared to trans-arterial chemoembolization for huge HCC [14]. Indeed, the effectiveness of TACE in patients with huge HCC may be impaired by the presence of extrahepatic collaterals that makes difficult to achieve complete tumor embolization [15]. Even in cases with type I and II portal vein tumor thrombus, LR has been reported to be superior to TACE [16].

International guidelines have officially approved the use of laparoscopy for HCC treatment, in the light of less intraoperative blood loss, fewer complications, faster postoperative recovery, and equivalent long-term outcomes than open liver resection (OLR), as well as a decreased risk for postoperative decompensation in fragile cirrhotic patients [17–20]. However, several limitations to the universal adoption of LLR for HCC still exist. In particular, the outcomes of laparoscopic liver resection (LLR) for huge HCC are still controversial.

In this scenario, we retrospectively analyzed the clinical and oncologic outcomes after LLR for patients with huge HCC.

## Materials and methods

### Patients and data

Data from consecutive patients undergoing LR for huge HCC from January 2003 to June 2022 at a tertiary referral HPB center (Seoul National University Bundang Hospital, South Korea) were retrospectively collected from a prospectively established database.

The inclusion criteria were as follows: male or female patients aged > 18 years, at least one nodule with histopathological confirmation of HCC larger than 99 mm, no extrahepatic metastasis, no tumor thrombus in portal vein or other major vessels, adequate future remnant liver volume according to current literature [13]. The exclusion criteria were as follows: liver resection extended to other abdominal organs other than gallbladder, histological finding of combined HCC–CCA.

According to the different surgical approach, patients were divided into two cohorts: the LLR group and the OLR group. Primary endpoints were the perioperative outcomes, while secondary endpoints were the long-term oncological outcomes.

This study was conducted according to the Strengthening and Reporting of Observational Studies in Epidemiology (STROBE) guidelines of the EQUATOR network [21]. Informed consent was obtained prior to every surgical procedure. The study was conducted in accordance with the principles of the Declaration of Helsinki and approved by the Institutional Review Board (B-2407-912-101).

### Preoperative management

Patients ‘management was decided by the institutional multidisciplinary team meeting including hepatologists, oncologists, and radiologists. Preoperative evaluations were similar in both groups and included routine blood tests, liver function, coagulation examinations, serum tumor markers, ICG clearance tests, and triphasic enhanced computed tomography (CT) and/or magnetic resonance imaging (MRI). The definition of resectable HCC was based on multidisciplinary team decision, according to comprehensive evaluation of liver function test, ICG clearance test, remnant liver volume, and liver compensation status.

After surgeons fully informed patients about the pros and cons of the two approaches, the final decision was made by surgeons’ and patients’ preferences.

### Surgical procedures

The laparoscopic procedures have been described in detail elsewhere [22–24]. The proximity of major vessels with the subsequent risk of ischemia of the remnant liver or R1 resection was an important factor to decide if a major liver resection was needed. Briefly, patients under intravenous total anesthesia were positioned in supine position, with the primary operator standing between patients’ legs, and the assistant and scopist standing on his sides. Carbon dioxide pneumoperitoneum was established with a pressure of 12–14 mmHg. A 12 mm port was used for the laparoscope, whereas two 12 mm ports and two 5 mm ports were inserted under vision and applied for the operation. Laparoscopic ultrasonography was routinely performed to confirm the positions of tumors, prevent the omissions of additional lesions, and guide the transection lines. An extracorporeal Pringle maneuver was prepared to eventually help controlling blood loss. The liver parenchyma was transected by a combination of a harmonic scalpel (Ethicon, Endo-Surgery, USA) and a laparoscopic cavitron ultrasonic surgical aspirator (CUSA, Integra-France) or Ligasure (Medtronic, USA). Intraparenchymal vascular and biliary vessels were secured by clips or sutures. The specimen was placed into a retrieval bag and extracted through a suprapubic incision. After hemostasis, a drainage tube was routinely placed near the surgical bed.

For the open procedure, a reverse L-incision was conducted with patients who underwent the same anesthesia in the supine position. The operating procedure was similar to LLR, and CUSA or clamp crushing was used as the main method for liver parenchyma transection.

### Postoperative management and follow‑up

Postoperative follow-up data were analyzed. Postoperative complications were classified according to the Clavien–Dindo classification [25]. PHLF, post-hepatectomy bile leakage (PHBL), and post-hepatectomy hemorrhage (PHH) were diagnosed and classified according to the International Study Group of Liver Surgery (ISGLS) guidelines [26–28]. Ascites was defined according to the International Ascites Club definition [29]. Surgical procedure was classified according to Brisbane classification [30].

All patients were examined in outpatients’ clinics within one month after discharge, undergoing clinical, biological, and imaging evaluations every 3 months after discharge for the first 2 years, according to the oncological protocols. Following controls were scheduled every 12 months if no relapse was found. In case of tumor recurrence, the case was re-examined by a multidisciplinary team (MDT) with the aim of carrying out curative treatment as much as possible. First therapeutical strategy for localized recurrent HCC was repeat hepatectomy, according to previous literature that have showed the same OS and DFS as primary liver resection [31]. In case liver resection was not indicated because of liver, as well as because of tumor or patient status, other locoregional therapies represented the second choice.

### Statistical analysis

Continuous data were expressed as mean and standard deviation (SD) or median and interquartile range (IQR), depending on whether they had a normal distribution or not. Group comparisons were performed using Student’s *T* test or Wilcoxon’s rank test, depending on the distribution of the variable. Categorical data were expressed as frequencies and associated percentages. Comparisons between groups were performed using Pearson’s chi-squared test or Fisher’s exact test, depending on the expected value of the variable of interest. Overall and recurrence-free survival analyses were performed using the Kaplan–Meier method to calculate the median and 95% confidence interval (CI), and comparisons were performed using the log-rank method. The median follow-up was analyzed using the reverse Kaplan–Meier method.

All statistical analyses were performed using SPSS software version 28.1 (IBM SPSS Inc. Chicago, IL).

## Results

### Patients and tumor characteristics

**Fig. 1:**
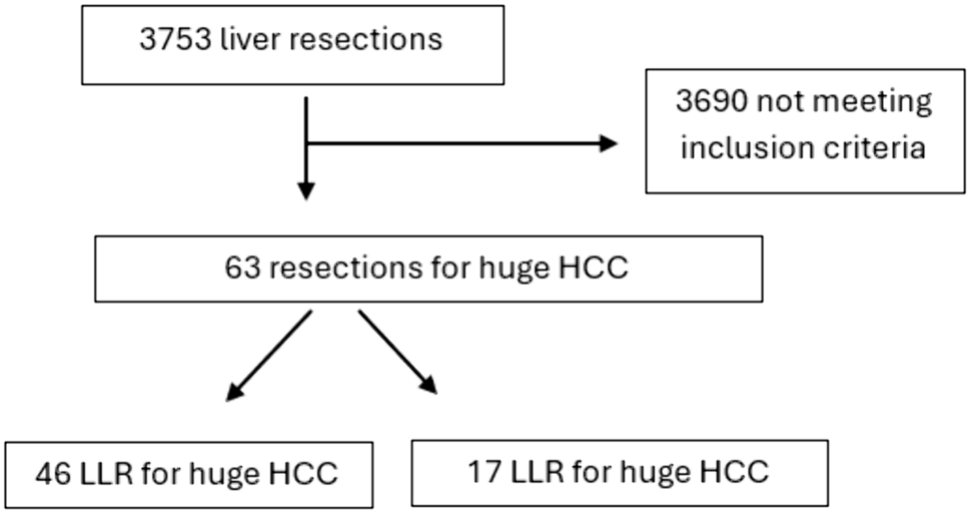
Study flow chart. *HCC* hepatocellular carcinoma, *LLR* laparoscopic liver resection, *OLR* open liver resection

During the study period, the institutional database included 3799 liver resections performed at Seoul National University Bundang Hospital. After removing lesions smaller than 10 cm and not matching the inclusion criteria, 63 patients were included in the study: 46 on the open liver resection (OLR) group and 17 in the laparoscopic liver resection (LLR) group (Fig. 1).

Mean age was 58.1 (± 13.3), with 12.6% of females (*n* = 8), and a median BMI of 24.1 (IQR = 3.8). Right hepatectomy was performed in 28 patients (44.5%), left hepatectomy in 10 patients (15%), a right trisectionectomy in 6 cases (9%), a mesohepatectomy in 4 cases (6.2%), a sectionectomy in 8 cases (12.6%), a bisegmentectomy in 2 patients (3.1%), a segmentectomy in 1 case (1.5%), and a wedge resection in 2 cases (3.1%).

No significant differences were found between the two groups in all the preoperative characteristics (Table 1).

**Table 1:** Preoperative patients’ and tumors’ characteristics

	Total (63)	OLR (46)	LLR (17)	*p*-value
Age, mean (SD)	58.1 (± 13.3)	59.2 (13.9)	55.2 (11.6)	0.30
Sex—female, *n* (%)	8 (12.6)	6 (13)	2 (11.8)	0.89
BMI, median (IQR)	24.1 (3.8)	23.9 (3.4)	25.1 (2.9)	0.07
Underlying liver cirrhosis, *n* (%)	28 (44.5)	20 (43.5)	8 (47.1)	0.80
Child, *n* (%)				0.91
A	23	17 (85)	6 (75)	
B	5	3 (15)	2 (25)	
MELD, median (IQR)	7.3 (1.3)	7.3 (2)	7.5 (1.1)	0.41
Etiology of underlying hepatopathy*, *n* (%)				0.31
HBV	48	26 (56.5)	12 (70.6)	
HCV	1	1 (2.2)	0 (0)	
Alcoholic	13	10 (21.7)	3 (17.7)	
Metabolic	11	9 (19.6)	2 (11.7)	
ASA score, *n* (%)				0.51
2	40	28 (60.9)	12 (70.6)	
3	20 (31.7))	15 (32.6)	5 (29.4)	
4	3 (4.7)	3 (6.5)	0 (0)	
Charlson comorbidity index, median (IQR)	4 (3)	4 (3)	4 (3)	0.90
HCC size, median (IQR)	115 (31)	118.5 (40.5)	104.7 (27.5)	0.07
AFP, median (IQR)	64.1 (988)	41.5 (556.7)	306 (1203)	0.17
Major resection	47 (74.6)	37 (80.4)	10 (58.8)	0.08
Type of resection, *n* (%)				0.10
Wedge	2 (3.1)	1 (2.2)	1 (5.9)	
Segmentectomy	1 (1.5)	0 (0)	1 (5.9)	
Bisegmentectomy	2 (3.1)	1 (2.2)	1 (5.9)	
Right anterior sectionectomy	1 (1.5)	1 (2.2)	0 (0)	
Right posterior sectionectomy	2 (3.1)	1 (2.2)	1 (5.9)	
Left lateral sectionectomy	5 (7.5)	3 (6.5)	2 (11.8)	
Mesohepatectomy	4 (6.2)	3 (6.5)	1 (5.9)	
Left hepatectomy	10 (15.8)	8 (17.4)	2 (11.8)	
Right hepatectomy	28 (44.5)	21 (45.7)	7 (41.2)	
Extended right hepatectomy	6 (9)	5 (10.8)	1 (5.9)	

*SD* standard deviation, *IQR* interquartile range, *MELD* model for end stage liver disease, *HBV* hepatitis B virus, *HCV* hepatitis C virus, *ASA* American society of Anesthesiologists, *AFP* alpha fetoprotein

*12 patients presented more than 1 etiology

### Postoperative outcomes

Intraoperative results showed no differences between OLR and LLR in mean operative time (287.5 ± 146 vs. 290 ± 250, respectively; *p* = 0.69), median estimated blood loss (800 vs 680, respectively; *p* = 0.53), and rate of intraoperative transfusions (41% vs 23.5%, respectively; *p* = 0.11).

Similarly, postoperative results showed no differences in the rate of PHLF (6.5% vs. 5.9%, respectively; *p* = 0.92), severe postoperative complications (8.7% vs. 5.9%, respectively; *p* = 0.71), and in-hospital mortality (2.2% vs. 0%, *p* = 0.54). All perioperative outcomes are found in Table 2.

**Table 2:** Perioperative outcomes

	OLR (46)	LLR (17)	*p*-value
Operative time, mean (SD)	287.5 (146)	290 (250)	0.69
Estimated blood loss, median (IQR)	800 (738)	680 (875)	0.53
Intraoperative transfusions, *n* (%)	21 (41)	4 (23.5)	0.11
R1 resection, *n* (%)	3 (6.5)	1 (5.9)	0.92
Postoperative complications, *n* (%)	20 (43.5)	7 (41.2)	0.87
Severe postoperative complications, *n* (%)	4 (8.7)	1 (5.9)	0.71
PHLF, *n* (%)	3 (6.5)	1 (5.9)	0.92
Length of stay, median (IQR)	9 (5)	8 (7)	0.79
In-hospital mortality, *n* (%)	1 (2.2)	0 (0)	0.54

*SD* standard deviation, *IQR* interquartile range, *PHLF* post-hepatectomy liver failure

### Survival analysis

**Fig. 2:**
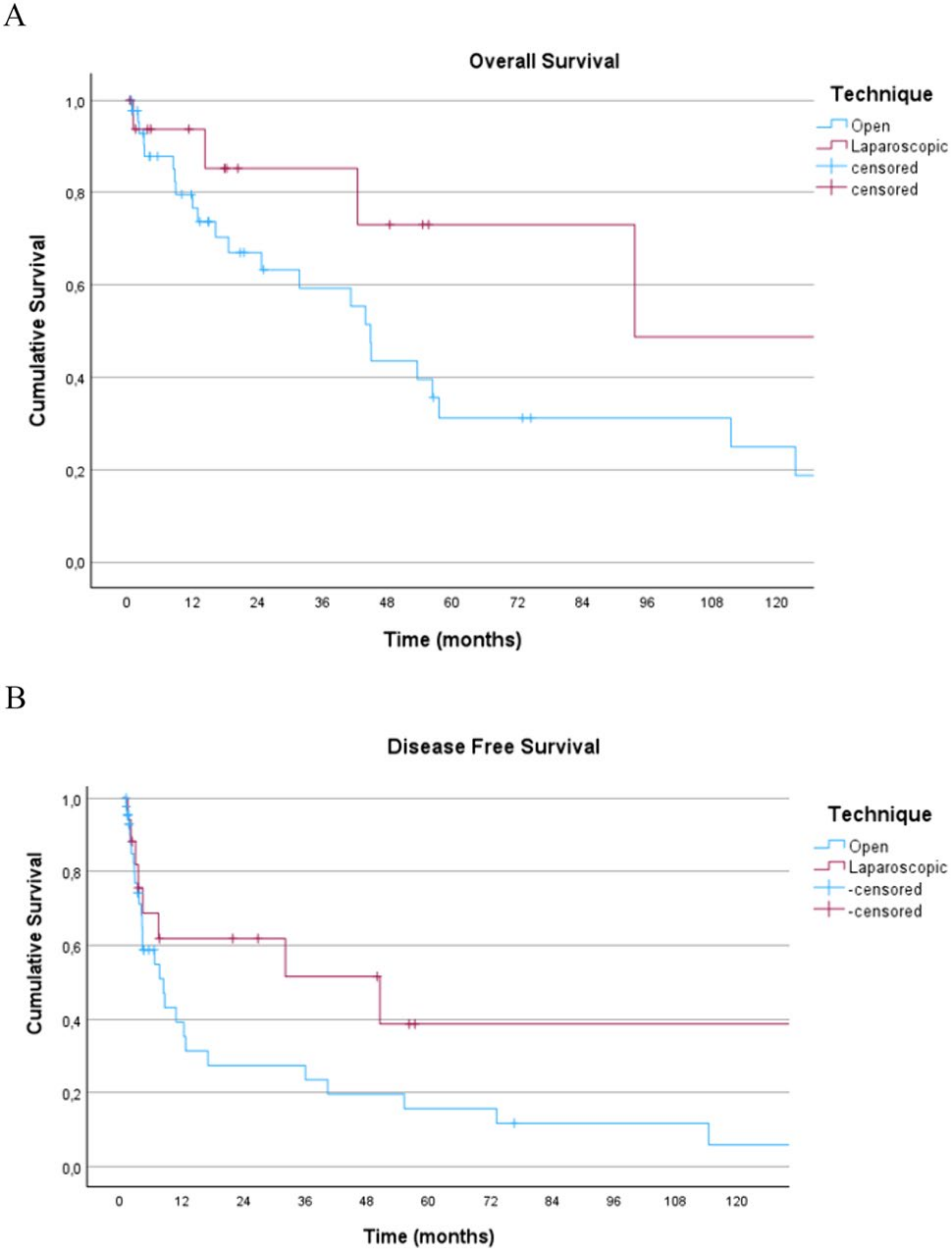
Overall survival (**A**) and disease-free survival (**B**) curves for patients with huge (>10cm) HCC undergoing laparoscopic liver resection (red) versus open liver resection (blue)

After a median follow-up of 48.4 (95% CI 8.9–86.8) months, there were no statistically significant differences in 3 years OS (59.3 ± 8.7 months vs. 85.2 ± 9.8 months) and 5 years OS (31.1 ± 9 months vs. 73.1 ± 14.1 months), after OLR and LLR, respectively (*p* = 0.10) (Fig. 2a).

Similarly, both 3 years DFS (23.5% ± 8.1 months vs. 51.6 ± months) and 5 years DFS (15.7 ± 7.1 months vs. 38.7 ± 15.3 months) were similar in the open and laparoscopic groups, respectively (*p* = 0.13) (Fig. 2b).

## Discussion

This was the largest study to deal with LLR for huge HCC, as well as the first one to specifically compare the short- and long-term results of the open and laparoscopic group for huge HCC. Our data showed that LLR may be safe and feasible in selected cases of huge HCC.

Many studies have previously reported that hepatic resection is the best available option for HCC larger than 10 cm, when compared to other therapeutic strategies [32]. Long-term recurrence is the main problem to face in these patients, and several prognostic risk factors have been identified, such as T4 status, macrovascular portal invasion, and the use of intraoperative transfusion by Yamashita and al., or serum alpha fetoprotein ≥ 100 ng/mL, hypermetabolic uptake on positron emission tomography, satellite nodules, and microvascular invasion by Hwang et al. [33]. In case of recurrence, timely and aggressive treatment is able to significantly improve long-term survival of HCC patients, with recurrent surgery always to be chosen in the optic of a hierarchic strategy and a personalized management of HCC patients [34, 35]. Within the entire cohort of 63 patients treated in our institution since the start of the HPB program, the 3 years OS was 65.8 (± 7.2) and the 5 years OS was 40% (± 8.5), while the 3 years and the 5 years DFS were 32.8 (± 7.4) and 23.2 (± 7). Such encouraging results are in line with previous literature and confirm the leading role of surgery for huge HCC patients. Furthermore, despite not showing statistically significant differences in OS between OLR and LLR groups, the OS was twice as high after LLR than after OLR, potentially reflecting clinically relevant differences. Further appropriated studies should investigate this aspect.

Regarding the role of LLR for the surgical treatment of HCC, many previous meta-analyses and propensity score matched studies have reported reduced bleeding, shorter hospital stays, and fewer postoperative complications, without affecting long-term results [19, 20, 36]. On these bases, previous consensus meetings have stated its safety and effectiveness in selected cases [37, 38]. Nonetheless, there are still some scenarios in which the role of LLR is still debated, such as for huge HCC, multiple HCC, or difficult located HCC [39].

There are several reports concerning laparoscopic hepatectomy for large liver cancer and these confirm that it can be performed safely, although the operative time is extended [40, 41]. Indeed, Goh et al. have reported that tumor size does not affect both short- and long-term outcomes [42]. However, laparoscopic liver resection for large liver tumors is technically challenging and is currently performed only by experienced surgeons in referral HPB centers. The difficulty of LLR for large tumors is due to the limited surgical view, together with the limited possibility of handling the underlying fibrotic or cirrhotic liver, and the proximity to blood and biliary structures [3]. Indeed, tumor size is one of the main parameters of the most used difficulty scores for LLR, and an interesting recent study by Xiaocui et al. reports a correlation between technical difficulty and long-term results after minimally invasive liver resection [43].

In this scenario, reporting our real-life data about the outcomes of LLR for huge HCC may add important evidence about its safety and effectiveness. There were no significant differences in both intraoperative and postoperative outcomes, including in-hospital, short-term, and long-term survival. Similarly, also the rate of R0 resection was similar. It is interesting to note that there were no differences also in the operative time, despite previous studies reporting longer operative time [44]. Probably, the surgeons’ and center experience play a key role in this aspect, as previously reported in literature [45]. Another interesting result was the rate of blood transfusions, that was almost double in the OLR when compared to LLR, despite non-significant (41% vs. 23.5%, *p* = 0.11). Further studies may focus on such aspect, given the prognostic importance of blood transfusions during liver resection for HCC [46, 47].

Furthermore, our population included all consecutive cases of huge HCC undergoing surgery at Seoul National University Bundang Hospital since the start of the HPB program. This reflects the real everyday life scenario in a HPB referral center, but, on the other hand, the results of the LLR case may even be partially influenced by the learning curve effect, given the progressive increase in number and technical difficulty of laparoscopic liver procedures in our center, which is notoriously accompanied by an improvement in the results [45]. Thus, results after LLR may be even better. Further studies may clear such aspect.

This study has some limitations. Firstly, its retrospective and single-center nature which is liable to selection bias. Nonetheless, to reduce selection bias, all consecutive patients meeting the selection criteria were included. Secondly, the small sample size that may affect the statistical results, due to the reduced statistical power that could make it difficult to achieve statistical significance (despite statistical significance is different from clinical significance) [48]. Indeed, it is well known that the absence of evidence is not evidence of absence, and results should not be misinterpreted in this sense [49]. When the question is if whether the absence of evidence is a valid enough justification for changing clinical practice, we must contextualize the clinical scenario. In our case, we included patients who already underwent LLR for huge HCC, as already reported in other centers, and, since patients with huge HCC are not always eligible for surgery, it is difficult to include larger sample size. We do not suggest changing clinical practice based on our results, but we believe it is important to report first available results from everyday practice in referral centers, in order to build further studies. Furthermore, we believe it is important to focus on how patients with huge HCC may be selected for LLR in referral centers. To this aim, a future strategy may be to design a multicenter study, but with the following risk of additional heterogeneity. This is still the largest monocentric series of LLR for huge HCC, so far. Thirdly, the patients were not matched according to the patients’ and tumors’ characteristics.

## Conclusion

Laparoscopic liver resection for giant tumors (larger than 10 cm) may be safely performed in selected cases in referral centers. Such results need to be confirmed by further studies with larger sample size.
